# Activation of Estrogen-Responsive Genes Does Not Require Their Nuclear Co-Localization

**DOI:** 10.1371/journal.pgen.1000922

**Published:** 2010-04-22

**Authors:** Silvia Kocanova, Elizabeth A. Kerr, Sehrish Rafique, Shelagh Boyle, Elad Katz, Stephanie Caze-Subra, Wendy A. Bickmore, Kerstin Bystricky

**Affiliations:** 1 Laboratoire de Biologie Moléculaire Eucaryote, Université de Toulouse - UPS, Toulouse, France; 2 LBME, CNRS, Toulouse, France; 3 The Breakthrough Breast Cancer Research Unit, Edinburgh, United Kingdom; 4 Medical Research Council Human Genetics Unit, Institute of Genetics and Molecular Medicine, University of Edinburgh, Edinburgh, United Kingdom; The Babraham Institute, United Kingdom

## Abstract

The spatial organization of the genome in the nucleus plays a role in the regulation of gene expression. Whether co-regulated genes are subject to coordinated repositioning to a shared nuclear space is a matter of considerable interest and debate. We investigated the nuclear organization of estrogen receptor alpha (ERα) target genes in human breast epithelial and cancer cell lines, before and after transcriptional activation induced with estradiol. We find that, contrary to another report, the ERα target genes *TFF1* and *GREB1* are distributed in the nucleoplasm with no particular relationship to each other. The nuclear separation between these genes, as well as between the ERα target genes *PGR* and *CTSD*, was unchanged by hormone addition and transcriptional activation with no evidence for co-localization between alleles. Similarly, while the volume occupied by the chromosomes increased, the relative nuclear position of the respective chromosome territories was unaffected by hormone addition. Our results demonstrate that estradiol-induced ERα target genes are not required to co-localize in the nucleus.

## Author Summary

Whether co-regulated genes relocalize in a coordinated fashion to a shared nuclear space is a matter of considerable interest and debate. We investigated the spatial organization of estrogen receptor alpha (ERα) target genes in three human breast epithelial cell lines, human epithelial cells (HMEC), the MCF10A normal-like diploid cell line, and the MCF-7 aneuploid tumor cell line. Nuclear positions of a subset of genes in the absence of hormone and upon addition of estradiol were assessed quantitatively using 2D and 3D in situ hybridization techniques. In our two laboratories, we find that *TFF1* and *GREB1* are distributed in the nucleoplasm with no particular relationship to each other. Distances between homologous and heterologous alleles of these genes and the relative nuclear position of their respective chromosome territories 2 and 21 was, contrary to a previous report, unaffected by transcription activation and hormone addition. Similar results were obtained with ERα target genes *PGR* and *CTSD* on chromosomes 11. Even in the anti-estrogen resistant LCC9 cell line, *TFF1* and *GREB1* and the two *TFF1* alleles remained separated after exposure to estradiol. Our results thus demonstrate that estradiol-induced genes are not required to co-localize or interact in trans or in cis.

## Introduction

Chromatin organisation in the vertebrate nucleus is non random: chromosomes adopt preferential positions with regard to the centre or edge of the nucleus and genes adopt preferential positions with regard to their own chromosome territory [1]. Moreover, preferential long-range associations have been found between loci, mainly in cis [2], [3] but also in trans [4]–[9]. Many of these associations have been suggested to be of functional significance for gene expression, either through the trans-interaction of genes and regulatory elements [4], [8], through the trans-sensing of homologous alleles prior to X chromosome inactivation [5], [9] or by the co-localisation of genes at the same transcription factory [7].

An instance of rapid and directed inter-chromosomal interactions has recently been reported for estrogen receptor α (ERα) target genes in primary human mammary epithelial cells (HMEC) and in a breast cancer cell line (MCF-7) [10]. ERα is a nuclear receptor that, in response to stimulation by 17β estradiol (E2), regulates gene expression by binding both promoters and more distal sites that may be long-range enhancers [2], [11]–[15]. E2 bound ERα accumulates in numerous nuclear foci [16], [17] which raises the possibility that there might be associations in the nucleus between multiple ERα binding sites, in cis and in trans. Activation of gene expression by ERα involves extensive chromatin remodelling mediated by the recruitment of histone modifying enzymes and nucleosome remodelling complexes [18]. Moreover, molecular motors such as dynein light chain (DLC1) have been reported to bind to ERα and to the promoters of ERα-responsive genes to potentiate their transcription [19], a dynactin component binds and modifies the function of ERα [20] and the microtubule network has also been implicated in ERα action [21]. These observations raise the possibility that directed long-range motion in the nucleus might be involved in ERα function.

Indeed, the rapid (within 1 hour) and directed long range movement of estrogen responsive genes reported after E2 exposure, was reported to be dependent on nuclear actin/myosin [10]. In particular, inter-chromosomal interactions detected by chromosome conformation capture (3C), and nuclear co-localisation revealed by fluorescence in situ hybridisation (FISH), were described between alleles of some estrogen inducible genes. More surprisingly, the movement was restricted to the gene loci concerned and involved rapid repositioning of the genes' chromosome territories within the nucleus. The estrogen-inducible genes that apparently showed this inter-chromosomal “kissing” [22] were *TFF1* (also known as pS2) on chromosome 21 and *GREB1* on human chromosome 2. Within 60 minutes of E2 addition to cells that had been grown in the absence of steroids, these genes were activated in ERα–positive MCF-7 cells and “monoallelic” and “biallelic” heterologous associations between *GREB1* and *TFF1* and between chromosomes paints for chromosomes 2 and 21 were reported, both in HMEC and MCF-7 cells [10].

Importantly, ERα activates the expression of these genes through *de novo* recruitment of RNA polymerase II (RNAPII), rather than, as is apparently the case for most ERα-responsive genes, through regulation of the phosphorylation state of RNAPII pre-loaded at the promoter [23]. Hence, it is possible that the reported nuclear co-localisation of these ERα-responsive genes represents their recruitment to a shared nuclear compartment that facilitates gene expression, such as transcription factories or splicing factor-enriched nuclear speckles [24].

Whilst there are other reported instances of rapid gene and locus motion within the nucleus [1], including an example where nuclear actin/myosin is involved [25], the view of rapid and extensive nuclear reorganisation induced by estrogen contrasts with other studies of the dynamics of specific loci or of whole chromosome territories. These have indicated that chromatin generally has limited mobility in mammalian cells. With the exception of the initial stages of G1 [26], chromatin motion appears to occur by constrained diffusion and is limited to a range of approximately 0.5 microns [27] over long periods (tens of minutes through to many hours) of interphase [27], [28]. Given this potential discrepancy, we sought to re-examine the nuclear organisation of *TFF1* and *GREB1* upon E2 stimulation in normal-like MCF10A and cancerous MCF-7 breast cancer cell lines and in primary HMECs. We found no evidence for nuclear co-localisation of *TFF1* and *GREB1* upon E2 stimulation in either situation and did not observe any directed, coordinated rearrangements of the chromosome 2 and 21 territories.

## Results

### Nuclear organisation of ERα–responsive genes in human mammary epithelial cells

The rapid inter-chromosomal co-localisation of estrogen responsive genes that are activated by addition of E2, and the nuclear repositioning of their chromosome territories, was reported in primary human mammary epithelial cells (HMECs) [10]. To reproduce this data, we prepared probes corresponding to the *GREB1* and *TFF1* loci and verified them, along with paints for chromosomes 2 and 21, by FISH to metaphase chromosomes from HT1080 and MCF10A cells, both of which have a near normal karyotype [29], [30]. The *GREB1* and *TFF1* probes mapped only to the expected positions at 2p25.1 and 21q22.3, respectively (Figure S1A and S1C), and each gave two distinct signals in the interphase nuclei of diploid cells (Figure S1B and S1D).

**Figure 1:**
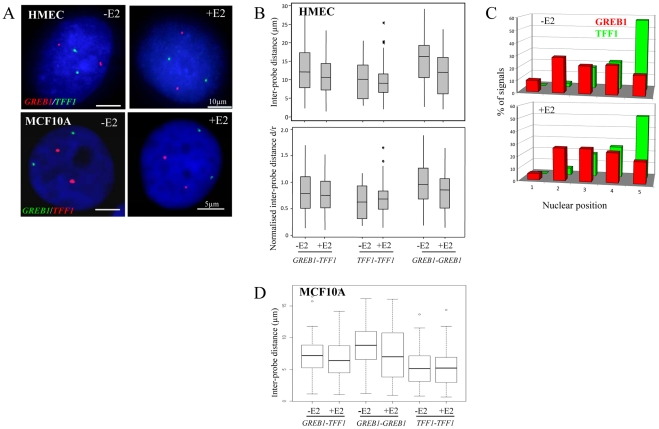
Nuclear organisation of *TFF1* and *GREB1* in diploid primary and tumor epithelial cells. (A) Interphase FISH with probes (red or green) for *TFF1* and *GREB1* on nuclei from HMEC and MCF10A cells in the absence of estradiol (-E2), or after 60 mins of E2 addition (+E2). Nuclei were stained with DAPI (blue). Scale bars, 10 µm and 5 µm. (B) Box plots showing; top: inter-probe distances (d in µm) and bottom: inter-probe distances (d) normalized to nuclear radius (r) between homologous or heterologous *TFF1* and *GREB1* alleles, as measured either by 3D FISH in HMEC nuclei grown in the absence (-E2) or after 1 hr of 100 nM E2 addition. Shaded boxes show the mean and 25–75 percentiles of the data. Asterisks indicate data points beyond the 95^th^ percentile. N = 50 cells. (C) Histograms showing the percentage of signals of the radial position of *TFF1* and *GREB1* alleles, before and after E2 addition, across 5 erosion shells placed between the edge (shell 1) and the centre (shell 5) of the nuclei. N = 50 cells. (D) Box plots showing inter-probe distances (µm) between homologous or heterologous *TFF1* and *GREB1* alleles, as measured by 3D FISH in MCF10A nuclei grown in the absence (-E2) or after 1 hr of 10 nM E2 addition. Asterisks indicate data points beyond the 95^th^ percentile. N = 50 cells.

These probes were then used on nuclei from two independent cultures of HMECs grown either in charcoal-depleted stripped media, i.e. in the absence of E2 (-E2), or after 60 mins of stimulation by 100 nM E2 (+E2). Nuclear positions were analysed by both 2D and 3D FISH. 2D FISH affords faster image analysis and although it slightly exaggerates interphase distances compared to 3D [31] it gave remarkably similar results on *TFF1*-*GREB1* distances compared to 3D analysis. Visual inspection of 3D FISH images revealed four distinct and separate hybridisation signals (two per gene) and so did not indicate any obvious co-localisation, either between homologous alleles of *TFF1* or *GREB1*, or between heterologous alleles of these genes (Figure 1A). We measured the interphase distances between all combinations of the hybridisation signals, and we also normalised each inter-probe distance (d) by the radius (r) of a circle of equal area to that of the nucleus to account for any changes in nuclear size as a consequence of E2 addition (Figure 1B). In 3D analysis, there was no significant difference in the normalised inter-probe distances before and after addition of E2, either for homologous alleles p≥0.2, or for the heterologous *TFF1*-*GREB1* probe pairs (p≥0.5). The mean separation between *TFF1*-*GREB1* alleles after E2 addition was 11 µm, with only 0.5% of measurements ≤1 µm.

The closest distances in HMEC nuclei were, in fact, recorded between the homologous *TFF1* alleles in the absence of E2 (p≥0.004). We considered this likely due to the fact that *TFF1* is located on the small acrocentric chromosomes 21, which are associated with the nucleolus and so constrained to a position within the small central volume of the nucleus. In contrast, chromosome 2, where *GREB1* resides, has a more peripheral nuclear location, affording the possibility of much larger nuclear distances between the homologues [32]. Indeed, analysis of the radial nuclear position of these two genes confirmed the more central nuclear position of *TFF1* alleles, with >50% of signals found in the innermost zone 5 of the nucleus (Figure 1C).

**Figure 2:**
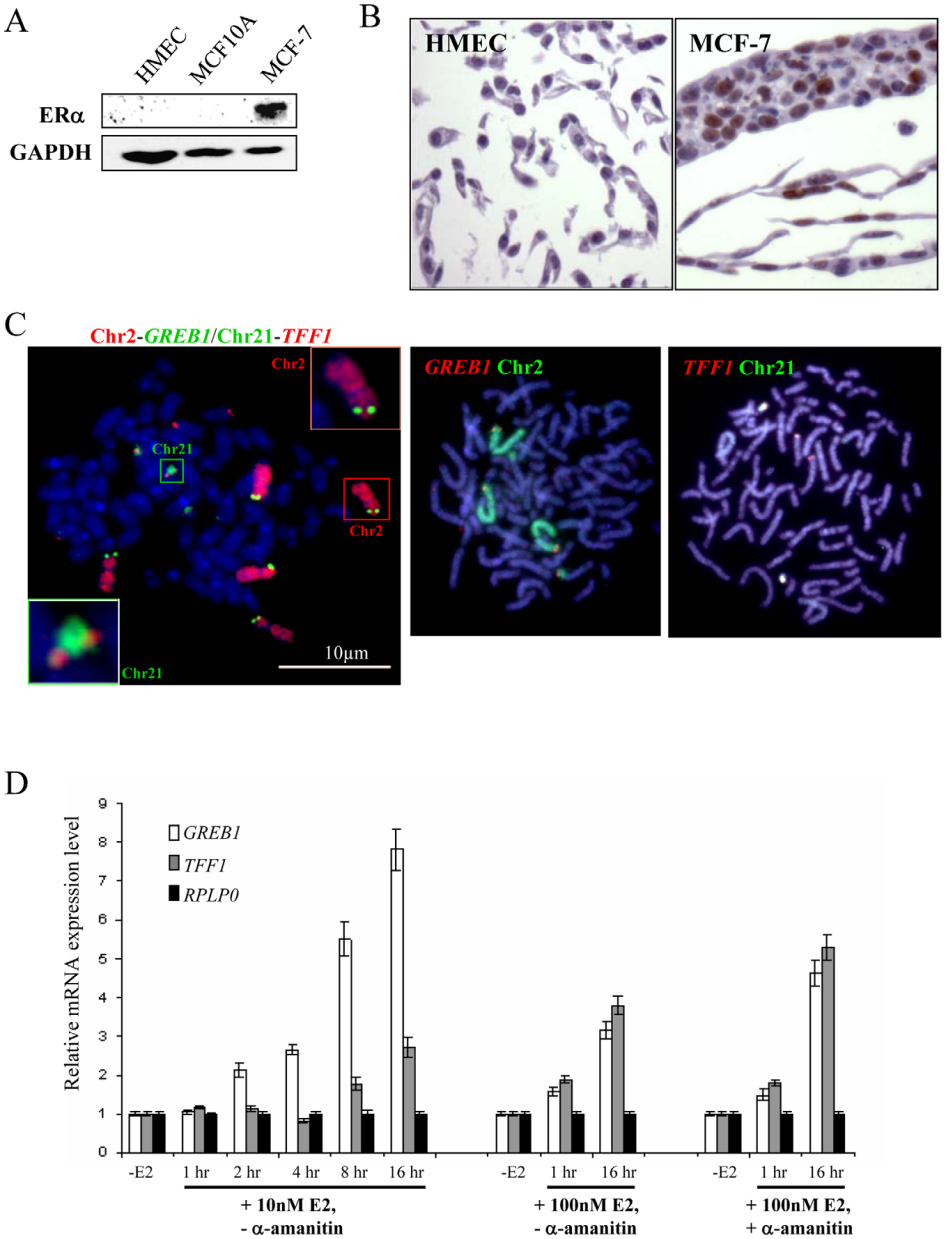
Expression and genomic arrangement of *TFF1* and *GREB1* in breast epithelial cells and MCF-7 breast cancer cells. (A) Western blot to detect protein expression of ERα and GAPDH in total cell lysate prepared from HMEC, MCF10A and MCF-7 cells. (B) Immunohistochemical staining of HMEC and MCF-7 cells with antibody that detects ERα (brown). (C) FISH with probes for *TFF1* and *GREB1*, and paints for chromosomes 2 and 21, on metaphase chromosomes prepared from MCF-7 cells. Bars, 10 µm. (D) Real-time qPCR analysis to detect the relative expression levels of *GREB1*, *TFF1* and a *RPLP0* control prepared from MCF-7 cells grown without E2 (-E2) and during 16 hr time courses in the presence of either 10 nM or 100 nM E2. Cells with (+) and without (−) pre-treatment with 2.5 nM α–amanitin were also examined.

The absence of nuclear co-localisation of ERα-responsive genes after E2 addition to cultures of HMECs cells is not that surprising, since these cell types are generally considered to have low or undetectable levels of ERα [33], [34]. Indeed, immunohistochemical staining revealed the absence of detectable ERα in the nucleus of these cells (Figure 2B) and the absence of ERα in these cells was confirmed by western blot (Figure 2A). Similarly, MCF10A cells, which are spontaneously immortalized human breast epithelial cells [35], and which have a normal diploid complement of *TFF1* and *GREB1* alleles (Figure S1C), also have no detectable ERα protein levels (Figure 2A). As in HMECs, no co-localisation of homologous or heterologous *TFF1* and *GREB1* alleles was seen in these cells (Figure 1A). The mean inter-probe distances measured after 3D FISH between heterologous alleles were ∼7 µm, with no changes after E2 addition (less than 1% at ∼1 µm before and after E2 stimulation) (Figure 1D). Similarly, the smallest inter-probe distances were found in MCF10A cells between homologous *TFF1* alleles, with ∼3% at less than 1 µm, in both the absence of E2 or after 1 h of E2 induction.

### Chromosomal rearrangements of regions carrying ERα–responsive genes in MCF-7 cells

We therefore tested the same *TFF1* and *GREB1* probes on ERα–positive (Figure 2A) MCF-7 breast cancer cells, which have also been reported to demonstrate rapid nuclear colocalisation of *TFF1* and *GREB1* upon E2 addition [10]. In this cell line between 4–6 hybridisation signals were seen for each of the gene probes on both metaphase and interphase MCF-7 preparations (Figure 2C). Similarly, chromosome paints indicated extensive rearrangement and gain of material from chromosomes 2 and 21 in independent isolates of these cells. There appear to be two normal copies of chromosome 2 that carry *GREB1* and one *GREB1*-carrying copy with additional material translocated onto the long arm of chromosome 2. A fourth copy of *GREB1* is on a small portion of chromosome 2p translocated onto another chromosome. Similarly, two copies of *TFF1* are on normal-looking chromosomes 21, with two or four additional copies translocated onto unidentified large chromosomes. Variable karyotypes have been described, by both cytogenetic and molecular methods, from MCF-7 cells grown in different labs at different times, but in all of them the cell line is highly aneuploid [36]–[41]. This genomic instability was recently confirmed by deep-sequencing [42] and our analysis is compatible with this.

Using quantitative RT-PCR (qRT-PCR) we confirmed that *TFF1* and *GREB1* expression was activated upon addition of E2 to MCF-7 cells. Activation of E2-responsive genes has been reported after addition of both 10nM [17], [18] and 100 nM [10], [23], [43] E2. Indeed, relative *TFF1* and *GREB1* mRNA levels in our MCF-7 cells increased 3 and 8 fold, respectively, after 16 h exposure to 10 nM E2 and 4 and 3 fold, respectively, using 100 nM E2 (Figure 2D). Moreover, steady-state levels of *GREB1* and *TFF1* mRNA increased 2 fold within 1 hr of 100 nM E2 addition, the time period during which co-localisation of these gene loci has been reported [10]. Several previous studies have also used a pre-treatment with the RNA polymerase II inhibitor α-amanitin before the addition of E2, in order to remove any active ongoing transcription from genes before their induction [10], [18]. In our analysis, α-amanitin did not change the initial response of cells to E2, but steady state levels of *TFF1* and *GREB1* mRNAs after 16 hrs E2 exposure were higher in the α-amanitin pre-treated cells, compared to untreated cells (Figure 2D).

### Absence of nuclear co-localisation of ERα–responsive genes in MCF-7 cells

**Figure 3:**
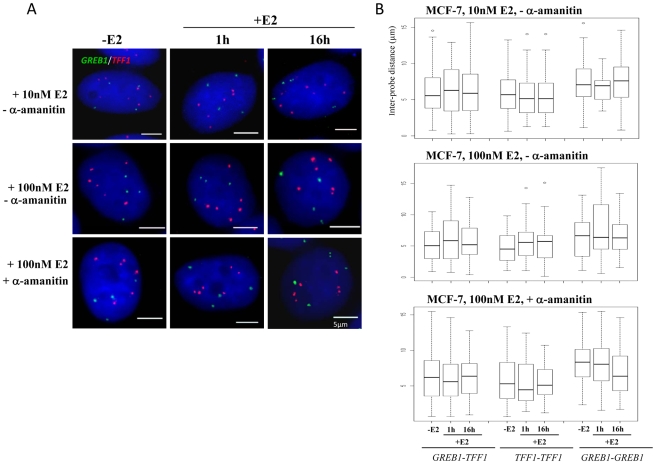
*TFF1* and *GREB1* nuclear organisation in ERα–positive MCF-7 breast cancer cells. (A) 3D Interphase FISH with probes for *TFF1* (red) and *GREB1*(green) on nuclei of MCF-7 cells grown either in the absence of estrogen (-E2), or 1 and 16 hrs after the addition of 10 nM (top row) or 100 nM (middle row) E2 (+E2). Bottom row: 1 h and 16 hrs of 100 nM E2 addition following pre-treament with 2.5 nM α-amanitin. Nuclei were stained with DAPI (blue). Scale bars  = 5 µm. (B) Box plots show inter-probe distances (µm) between homologous or heterologous *TFF1* and *GREB1* alleles, as measured either by 3D FISH in nuclei of MCF-7 cells grown in the absence of E2 (-E2) or 1 and 16 hr after the addition of 10 nM (top) or 100 nM (middle) E2. Bottom row: 1 h and 16 hrs of 100 nM E2 addition following pre-treament with 2.5 nM α–amanitin. Asterisks indicate data points beyond the 95^th^ percentile. N = 40 cells.

We did not observe co-localisation of *GREB1*-*TFF1* alleles in untreated or E2 treated MCF-7 cells (Figure 3A). We analysed the inter-probe distances (Figure 3B) and the normalised inter-probe distances (Figure S2A) between all possible homologous or heterologous pairs of gene signals in nuclei from MCF-7 cells grown in steroid-free media in the absence of E2, and then 1 and 16 hrs after the addition of either 10 nM or 100 nM E2. We scored >750 inter-probe distances of *TFF1-GREB1* alleles and found on average 5%<2 µm (2–11 measured distances per experiment with or without E2) and 2.5%<1 µm (1–3 distances per experiment). We never observed a single nucleus in which all possible *TFF1-GREB1* distances were <1 or 2 µm. Overall in ∼10% of nuclei one or two inter-probe distances <2 µm were observed. This proportion was highly variable (3–18%) from experiment to experiment since there were so few small distances. Such close proximity between two genes may thus be a transient and randomly occurring situation. In addition we also analysed the nuclear organisation of these genes in response to 100 nM E2 addition in cells which had been pre-treated with α-amanitin, the experimental conditions under which nuclear colocalisation of *TFF1* and *GREB1* has previously been reported 1 hr after E2 addition [10]. There was also no difference in the *TFF1*-*GREB1* distances before and after E2 addition (p>0.2). The average inter-probe distance in 100 nM E2 treated cells was 6 µm, and only 3% and 2% of distances between heterologous alleles were ≤1 µm in untreated or α-amanitin pre-treated MCF-7 cells, respectively. We did also not observe any changes in distances between *GREB1-GREB1* alleles (p>0.2) or *TFF1-TFF1* alleles (p>0.4) upon E2 stimulation.

**Figure 4:**
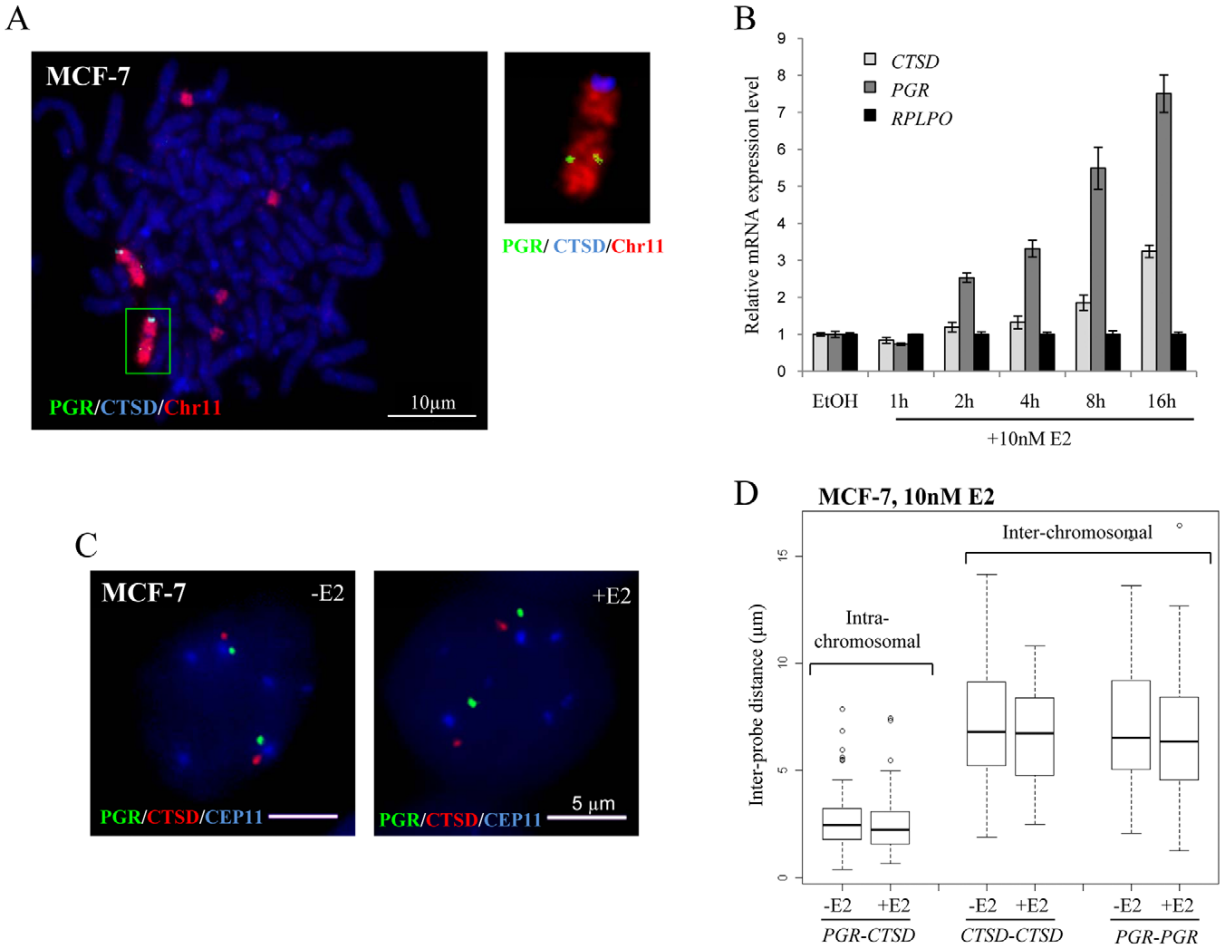
Localisation, expression, and nuclear organisation of *PGR* and *CTSD* in MCF-7 cells. (A) FISH with probes for *CTSD* (blue) and *PGR* (green) and paint for chromosome 11 (red) on metaphase chromosome spreads of MCF-7 cells. Scale bars  = 10 µm. (B) Real-time qPCR analysis to detect the relative expression levels of *PGR*, *CTSD* and a *RPLP0* control prepared from MCF-7 cells grown without E2 (-E2) and during 16 hr time courses in the presence of 10 nM E2. (C) 3D Interphase FISH with probes for *CTSD*(red), *PGR*(green) and the centromere of chromosome 11 CEP11 (blue) on nuclei of MCF-7 cells grown in the absence of estrogen (-E2) and 3 hrs after the addition of 10 nM E2 (+E2). Nuclei were counterstained with DAPI (blue). Scale bars  = 5 µm. (D) Box plots show inter- and intra-chromosomal distances (µm) between *PGR* and *CTSD* alleles, as measured by 3D FISH in nuclei of MCF-7 cells grown in the absence of E2 (-E2) and 3 hrs after the addition of 10 nM E2. Asterisks indicate data points beyond the 95^th^ percentile. N = 50 cells.

To determine whether the absence of nuclear colocalisation between *TFF1* and *GREB1* was an exception or a more general feature of estrogen induced transcription, we analysed the positioning of the progesterone receptor (*PGR*) and Cathepsin-D (*CTSD*) genes, both located on different regions of chromosome 11. Metaphase FISH indicated that both alleles of these genes reside on normal-looking chromosomes 11 in MCF-7 cells (Figure 4A). There are four additional translocation fragments that contain material from the centromere of chromosome 11 as reported by Bautista et al. [44], but these contain no additional copies of our genes of interest. Using qRT-PCR, we confirmed that *PGR* and *CTSD* expression was activated upon addition of E2 to MCF-7 cells (Figure 4B) with relative mRNA levels increasing 3 and 8 fold, respectively, after 16 h exposure to 10 nM E2.

As for the *GREB1*-*TFF1* alleles, we did not observe any change in nuclear separation for the *PGR*-*CTSD* alleles in untreated versus E2 treated MCF-7 cells (Figure 4C). Indeed, there was no difference in intra-chromosomal *PGR-CTSD*, or inter-chromosomal *PGR-PGR* or *CTSD-CTSD* distances before and after E2 (10 nM, 3 hrs) addition (p>0.5) (Figure 4D). As expected from the existence of chromosome territories, the average intra-chromosomal inter-probe distances were less than the inter-chromosomal ones, both before and after E2 addition (*PGR-CTSD*, 2.5 µm; *PGR-PGR*, 6.8 µm; *CTSD-CTSD*, 6.6 µm). Less than 1% of the inter-chromosomal and 10% of the intra-chromosomal probe distances were ≤1 µm. We did not observe any nuclei in which probe signals overlapped (<250 nm separation).

### Absence of nuclear co-localisation of ERα–responsive genes in cells with elevated TFF1

**Figure 5:**
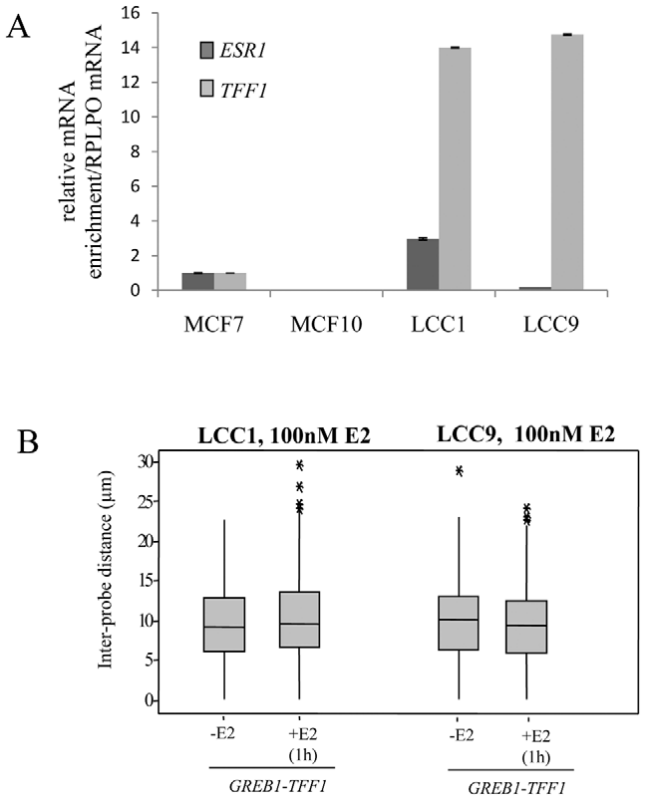
*TFF1* and *GREB1* nuclear organisation in LCC1 and LCC9 cells. (A) Real-time qPCR analysis to detect the relative expression levels of *ERα (ESR1)* and *TFF1* prepared from MCF-7, MCF10A, LCC1 and LCC9 cells grown in phenol red containing DMEM. (B) Box plots showing inter-probe distances (d) between heterologous *TFF1* and *GREB1* alleles, as measured by 2D FISH in nuclei of LCC1 or LCC9 cells grown in the absence of E2 (-E2) or after 1 hr in the presence of 100 nM E2. Asterisks indicate data points beyond the 95^th^ percentile. N = 50 cells.

In addition, we investigated the position of *GREB1* and *TFF1* genes in LCC1 and LCC9 cells which are clonal derivatives of the MCF-7 cell line [45], [46]. LCC1 is hormone independent for growth but still responsive to estrogen and anti-estrogens. LCC9 cells are resistant to anti-estrogens, fulvestrant and tamoxifen. Compared to MCF-7 cells *ESR1* mRNA levels are tripled in LCC1 cells and reduced to about 30% in LCC9 cells, yet both cell lines express very high baseline levels of *TFF1* (Figure 5A).

As for the parental MCF-7 cells, we found no evidence for nuclear co-localisation of *TFF1* and *GREB1* before or after E2 addition in either LCC1 or LCC9 cell lines (Figure 5B and Figure S2B). These results suggest that increased transcription rates of the *TFF1* gene do not promote interaction with another ERα-regulated gene.

### Absence of nuclear re-organisation of chromosome territories

**Figure 6:**
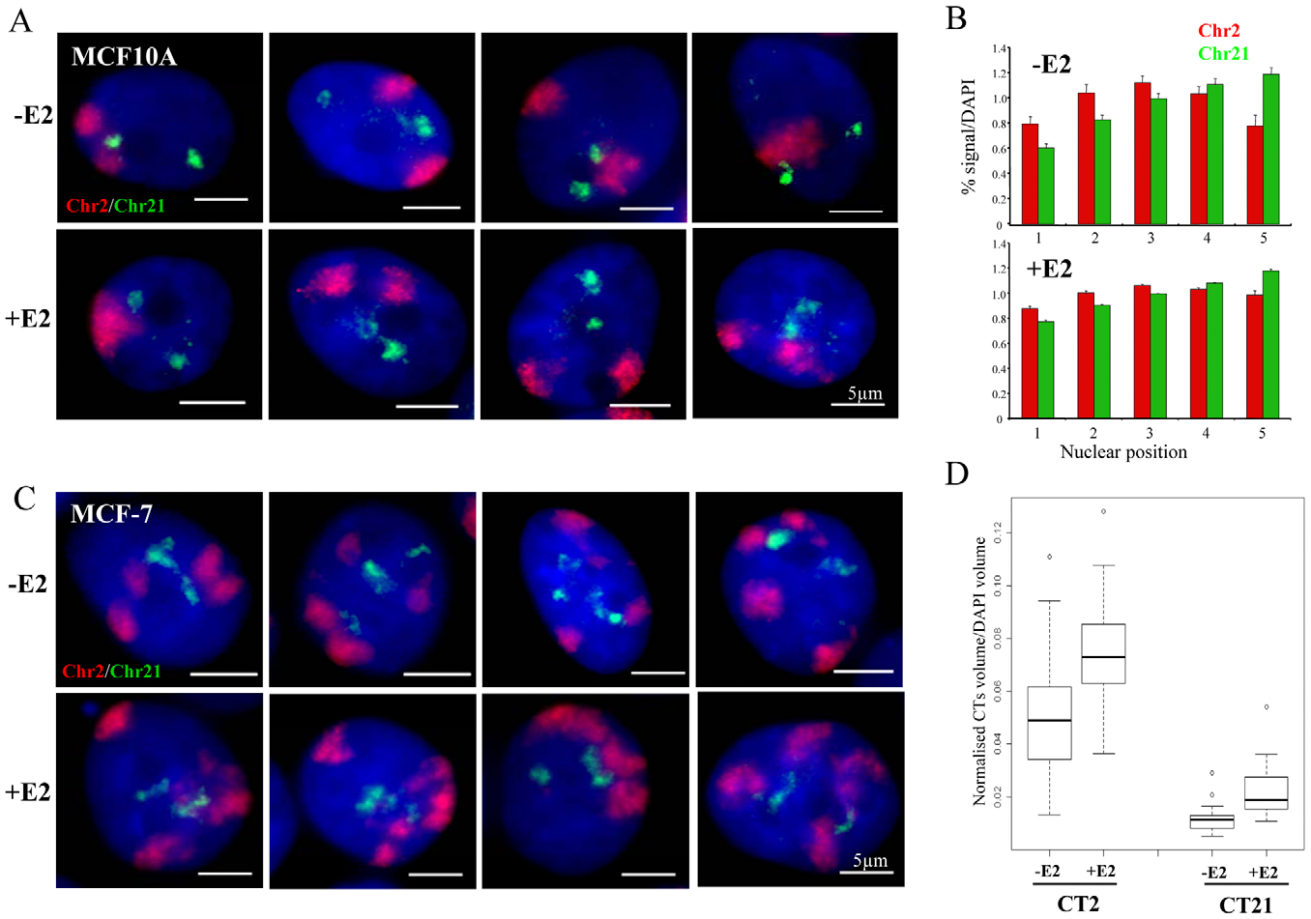
Chromosome territory organisation in MCF10A and MCF-7 cells. (A) 3D interphase FISH with chromosome paint to chromosome territory 2 (red) and 21 (green) on nuclei from MCF10A cells imaged in the absence of estrogen (-E2), or after 60 mins of 10 nM E2 addition (+E2). Nuclei were stained with DAPI (blue). Scale bars  = 5 µm. (B) Histograms showing the percentage of chromosome paint hybridization signal normalized to the percentage of DAPI signal, before and after E2 addition, across 5 erosion shells placed between the edge (shell 1) and the centre (shell 5) of the nuclei. N = 25−30 cells. (C) 3D interphase FISH with chromosome paint to chromosome territory 2 (red) and 21 (green) on nuclei from MCF-7 cells imaged in the absence of estrogen (-E2), or after 60 mins of 100 nM E2 addition (+E2). Nuclei were stained with DAPI (blue). Scale bars  = 5 µm. (D) Box plots show the percentage of chromosome paint hybridization signal (CT2 and CT21) normalized to the percentage of DAPI signal, before and after E2 addition. Asterisks indicate data points beyond the 95^th^ percentile. N = 30 cells.

Finally, we examined the relative nuclear position of the territories of chromosomes 2 and 21 by 3D FISH. In MCF10A cells, the two territories of chromosome 2 (Chr2) are frequently near the nuclear periphery, while, as expected, Chr21 localised near the nucleoli in a more central nuclear position (Figure 6A) [32]. In 40–50% of the untreated cells, Chr2 and Chr21 are adjacent to each other yet without significant overlap. Upon 1 h exposure to 10 nM E2, the relative positions of Chr2 and Chr21 and their general localisation in the nucleus did not vary from that observed in untreated cells. Notably, we did not observe an increase in cells in which Chr2 and Chr21 co-localised. We analysed the radial nuclear position of Chr2 and Chr21 and we confirmed more than 40% of Chr21 in the central nuclear space (shell 5) compare to the edge (shell 1) of the nuclei (Figure 6B). These observations are in good correlation with the results for *TFF1* genes (Figure 1C) that are located on Chr21.

We also examined the position of Chr2 and Chr21 in MCF-7 cells, where inter-chromosomal associations between the territories of these two heterologous chromosomes have been reported to be induced by E2 [10]. We did not observe any obvious significant overlap between Chr2 and Chr21 (Figure 6C, representative pictures are shown) upon E2 stimulation. However, the highly aneuploid and highly rearranged nature of these chromosomes in this breast cancer cell line (Figure 2C) makes it impossible to unequivocally differentiate the large number of chromosome territories and to objectively quantify their spatial relationships. Interestingly, we observed that after estradiol treatment both chromosome territories expanded. We examined the volume of chromosome territories and we detected ∼1.5 fold increase after E2 stimulation in MCF-7 cells (Figure 6D). We note that Chr2 and Chr21 are frequently neighboring each other, but this relative position did not change upon E2 addition and did not lead to colocalisation of *TFF1* and/or *GREB1* genes (Figure 3A).

## Discussion

The regulation of gene expression by elements located in cis, even when >1 Mb from the gene, is well accepted and increasingly well understood. In one case, this has been shown to be accompanied by co-localisation in the nucleus between the distant enhancer and the gene [47]. Because there is a degree of intermingling between chromosome territories [48] and because individual loci can loop out of their own chromosome territory [49], it is conceivable that a regulatory element could interact with a gene locus located on another chromosome. Such an interaction might be between homologous chromosomes when there is a need to establish differential expression states on these chromosome pairs, such as in X-chromosome inactivation (XCI) or imprinting. Whereas the transient pairing between X chromosomes during the establishment of XCI has been confirmed by two independent groups [5], [9], nuclear co-localisation between imprinted regions of autosomes has not been substantiated [50]–[52].

In vertebrate cells, even if reports of nuclear co-localisation between genes and regulatory elements in trans were to be verified [4], [8], [10], their functional significance remains unclear in the absence of direct genetic evidence for specific regulation in trans between heterologous chromosomes [53]. Such a deterministic view of nuclear organisation is also at odds with the non-heritable relative position of heterologous chromosomes. Although the disposition of chromosome territories in the nucleus is non-random, with a gene-density related radial organisation well described, it is also probabilistic in that the precise neighbourhoods of any particular chromosome change from one cell cycle to the next [28], [54]. This seems too loose an arrangement of chromosomes to ensure the dependable spatial juxtaposition of genes and regulatory elements in trans.

A less rigid spatial arrangement of genes in trans may come about through the sharing of multiple, but limited, nuclear compartments by genes that are active or inducible in a particular cell type. Such compartments might be rather general, for example transcription factories, or splicing factor enriched speckles [24], [55], [56], indeed it is suggested that the co-localised alleles of *TFF1* and *GREB1* in E2-treated breast epithelial cells are associated with the latter nuclear compartment [10]. Alternatively, such compartments may be specific to a particular pathway of regulated gene expression [57]. Nuclear hormone receptors have been reported to concentrate in discrete foci in the nucleus. In the case of the progesterone receptor, these foci do appear to correspond to sites of RNA synthesis, raising the possibility that they represent the specific nuclear sites of transcription of genes regulated by this hormone receptor [58], however, foci of the glucocorticoid receptor do not seem to correspond to sites where genes are expressed [59]. For ERα, the presence of the agonist ligand E2 results in rapid redistribution of the protein to discrete nuclear foci [16], [17]. Although these foci do correspond to a less mobile form of ERα - indicating binding, and they are the sites of recruitment of co-activators such as SRC-1, they do not significantly overlap with sites of transcription, calling into question whether they are the actual nuclear compartments for the activation of ERα-induced genes. Nor do these foci overlap with splicing factor enriched nuclear speckles [17], which are the nuclear structures where ERα-induced gene loci have been reported to co-localise [10].

E2-induced nuclear foci are numerous – there appear to be many 100 s or 1000 s of them [17] and their number is not dissimilar to the estimated range of E2-upregulated genes (∼100–700) or ERα binding sites (∼1000–3500) in the genome of MCF-7 cells [12], [14]. Therefore, it is not clear why up to 60% [10] of any two particular heterologous E2-induced genes (e.g. *TFF1*-*GREB1*) should co-localise at just one nuclear site, rather than each gene localizing at any of the many other ERα sites or splicing factor enriched speckles, perhaps with any of the other 100 s of E2-induced alleles. If this were a reflection of a pre-existing spatial proximity of the two genes in the nucleus, then one might rather expect to see preferential co-localisation of the two homologous alleles of *TFF1*, since we have shown (Figure 1B) that these alleles have a closer spatial proximity to each other, than to the *GREB1* alleles. We suggest that this reflects the location of *TFF1* on the small acrocentric chromosomes 21 that are constrained, through their nucleolar association, to the small central volume of the nucleus (Figure 1C). Indeed, a recent re-evaluation of reported pairing between imprinted alleles on human chromosomes 15, suggested that this was a secondary effect due to the convergence of the acrocentric chromosome 15 s at the nucleolus [52].

In normal and cancer breast epithelial cells, we can find no evidence for E2-induced nuclear co-localisation between the heterologous or homologous alleles of *TFF1* and *GREB1* (Figure 1, Figure 2, Figure 3, and Figure 5) or indeed for any allele combination of another pair of ERα-regulated genes, *PGR* and *CTSD* located in cis on the same chromosome as each other (Figure 4). Given the existence of chromosome territories as a major principle of nuclear organisation, this might have afforded increased opportunity for gene co-localisation in cis, albeit at long range. We cannot exclude that “chromosome kissing” between these genes is very transient and that we have just missed a critical time-window in our analysis, either before 1 hour of induction or between 1 and 16 hours, or that the increased steady state levels after the addition of E2 is the result of transcription from a very small proportion of alleles at any one moment in time [60]. However, *TFF1*-*GREB1* co-localisation has been reported as soon as 15 minutes after E2 addition and until 60 minutes after hormone addition, and precedes gene activation itself [10]. Similarly, ERα and its co-activators are bound to promoters of responsive genes within 30 minutes of E2 addition and are still there at 60 minutes [18], [43], [61]. Even at very long time periods (16 hours) after E2 addition, when we see maximal expression of *PGR*, *CTSD*, *TFF1* and *GREB1* in MCF-7 cells (Figure 2D and Figure 4B), we find no evidence for nuclear co-localisation of these genes (Figure 3B, Figure 4C). Moreover, our demonstration that HMECs do not express ERα (Figure 2A and 2B) and that MCF-7 cells are karyotypically abnormal and aneuploid for *GREB1*/Chr 2 and *TFF1*/Chr21 (Figure 2C, Figure 3A, and Figure 6C) precludes these cellular systems as having being appropriate models in which to record bi-allelic “kissing” of these loci and chromosomes in response to stimulation by estrogen.

In agreement with the notion that chromosome order established at the exit of mitosis remains stable throughout interphase [28], the relative nuclear position of the analysed chromosome territories was also unaffected by hormone addition in normal and cancerous cells. We noticed, however, that upon E2 treatment the nuclear volume occupied by the chromosomes increased about 1.5 fold. The association of agonist bound ERα with ∼100 s of target genes at numerous intranuclear sites causes decondensation [62] of chromatin simultaneously at all sites of activated transcription. This general chromatin decompaction thus supports our view that hormone stimulated gene activation occurs at multiple sites throughout the nucleoplasm.

Consistent with our findings, a recent genome-wide analysis, using a new CHIA-PET method, of long-range ERα-bound chromatin interactions in E2 stimulated MCF-7 cells [63] detected 689 intra-chromosomal interactions, but no validated inter-chromosomal interactions were found. Even if there were limitations in the coverage or interpretation of this data set, this experiment indicates that any specific ERα-mediated interactions between *TFF1*-*GREB1* [10], or indeed between any other functionally relevant combination of ERα targets on different chromosomes, occurs at a low frequency compared with intra-chromosomal interactions. Moreover, the intra-chromosomal interactions detected were mostly within a 100 kb size range, including those for *TFF1*, *GREB1*, *PGR* and *CTSD*, <1% encompassed distances of >1 Mb [63]. We therefore conclude that ERα does not generally mediate rapid interactions between distant target genes, either located >1 MB in cis (*PGR-CTSD*) or in trans (*TFF1-GREB1*), but that changes in chromatin and nuclear structure mediated by ERα are relatively local (generally within a 100 kb size range).

## Materials and Methods

### Cell culture and cell lines

Primary HMEC cells of luminal origin were isolated from normal breast tissue as previously described [64] and were maintained in CnT22 medium (CellnTEC) supplemented with 10% heat-inactivated fetal calf serum (FCS). The normal-like diploid human breast epithelial cell line MCF10A (ATCC purchase at July 2008 as a passage No. 97 and we used up to 10^th^ passages of MCF10A in all experiments) was maintained in Dulbecco's modified Eagle's medium F-12 (DMEM F-12) with Glutamax containing Mammary Epithelial Growth Supplement (MEGS), 10 ng/ml hEGF and 100 ng/ml cholera toxin.

Experiments on the human ERα–positive breast cancer cell line MCF-7 were conducted independently in both Toulouse and Edinburgh. In Toulouse, the cells were from ATCC, purchased at February 2008 as a passage No. 146 and used up to 10^th^ passages, maintained in DMEM F-12 with Glutamax containing 50 µg/ml gentamicin, 1 mM sodium pyruvate and 10% FCS. In Edinburgh MCF-7, as well as LCC1 and LCC9 cells were a gift of Bob Clarke (Georgetown University School of Medicine, Washington DC). These cells were maintained in DMEM without phenol red and supplemented with 5% L-Glutamine, 5% Penicillin/Streptomycin and 10% FCS. All cells were grown at 37°C in a humidified atmosphere containing 5% CO_2_.

To study the effects of 17β estradiol (E2) on ERα-target gene dynamics, cells were grown for 3 days in medium containing phenol red-free media supplemented with 5–10% charcoal-stripped FCS (-E2) and subsequently treated with 10 nM or 100 nM E2 (Sigma) for the indicated times. The cells synchronized by α-amanitin were pre-treated for 2 h with 2.5 nM α-amanitin, followed by washing and recovering for 1 h in normal steroid and phenol-red free media and then stimulated with 100 nM E2 for 1 h and 16 h.

### IHC

HMECs and MCF-7 cells were harvested by cell scraping and the cell pellets were mixed in 2% agarose/PBS. The pellet mix was left to cool and then paraffin-embedded using a Leica ASP300S automatic processor. Standard immunohistochemistry protocol was performed on 3 micron sections using the REAL EnVision mouse/rabbit kit (Dako), according to manufacturer's instructions. Antigen retrieval for ERα was performed using sodium citrate buffer (18 µM Citric Acid, 82 µM sodium citrate, pH 6.0). Anti-human ERα antibody (Vector Labs, VP-E613) was used at a dilution of 1∶50 for 1 h in room temperature.

### FISH

2D FISH on metaphase and interphase cells was as previously described [65]. In Toulouse, 3D FISH experiments were adapted from a previously protocol [66]. Cells were grown for 3 days on coverslips in DMEM without phenol red, containing 5% charcoal-stripped FCS, before addition of 10 nM or 100 nM E2 for the indicated times. Coverslips were washed twice with PBS, fixed in 4% paraformaldehyde (pFA)/PBS for 10 mins at room temperature and during the last minute 200 µl of 0.5% Triton X-100/PBS were added into 500 µl of 4% pFA. After fixation, the cells were washed three-times for 3 mins in 0.01% Triton X-100/PBS, incubated in 0.5% Triton X-100/PBS for 10 mins at room temperature and then incubated with 0.1 mg/ml RNase in 2xSSC for 1 hour at 37°C. After 3×10 mins washes in PBS, cells were incubated in 0.1 M HCl for 5 mins at room temperature, twice in 2xSSC for 3 mins and then equilibrated overnight in 50% formamide/2xSSC (pH = 7.2). MCF10A cells, with a large cytoplasm, underwent optional treatment with pepsin. In brief, equilibrated slides (kept in 50% formamide/2xSSC) were incubated in 2xSSC for 2 mins at room temperature, equilibrated in PBS for 3 mins at room temperature, incubated in 0.005% pepsin/0.01 M HCl pre-warmed to 37°C for 5 mins, incubated twice in 50 mM MgCl_2_ for 5 mins at room temperature, post-fixed in 1% pFA/PBS for 10 mins at room temperature, washed in PBS for 5 mins at room temperature, washed twice in 2xSSC for 5 mins and then returned into 50% formamide/2xSSC (pH = 7.2) for at least 1 hour before hybridization. Denaturation of the probes and target DNA is performed simultaneously at 85°C for 2 minutes.

In Edinburgh, cells were grown on Superfrost slides in the appropriate media, washed 3x in PBS before fixation in 4%pFa/PBS for 10 mins. After 3 further washes in PBS the cells were permeabilised in 0.5% Triton X-100/PBS for 10 mins and then washed 3x in PBS again. The slides were then incubated in 0.1 mg/ml RNase in 2xSSC for 1 hour at 37°C, washed again and then put through an ethanol series (70%, 90%, 100%) for 2 minutes each. The slides were warmed to 70°C in an oven for 5 minutes prior to denaturation in 70% deionised formamide, 2xSSC, pH 7.0 for 15 minutes at 80°C.

DNA from BAC clones (Toulouse) which include *TFF1* (RPCIB753I15619Q), *GREB1* (RZPDB737C102019D), *PGR* (RP11-788M5) or *CTSD* (RZPDB737F022085D) were directly labeled using nick translation (BioPrime DNA Labeling System, Invitrogen) by incorporation of fluorochrome-conjugated nucleotides Atto647N-dUTP-NT (Jena Bioscience) or ChromaTide AlexaFluor 488-5-dUTP (Molecular Probes). DNA from fosmid clones (Edinburgh) which encompass *TFF1* (G248P89501F6) or *GREB1* (G248P80076843) were nick-translated with biotin-16-dUTP or digoxigenin-11-dUTP. Chromosome paints were obtained from Genetix Ltd., UK (Toulouse) or from Cambio (Edinburgh). We used 100 ng labeled DNA probe or 100 ng chromosome paint together with 7 µg Cot-1 DNA and 5 µg sonicated salmon sperm DNA per slide. CEP11 (chromosome 11 alpha-satellite probe D11Z1) was obtained from MP Biomedicals.

### Microscopy and quantification

In Toulouse cells were examined by fluorescence microscopy using an Olympus IX-81 microscope, equipped with a CoolSNAPHQ camera (Roper Scientific) and imaged through an Olympus oil-immersion objective 100x PLANAPO NA1.4. Subnuclear position was captured on 21-image (200 nm step size) stacks and analysis of inter-probe distances between the centroid of each signal was performed using Metamorph software (Universal Imaging). Images were processed using Adobe Photoshop 9.0.2.

In Edinburgh, 2D specimens were examined with a Zeiss Axioplan II microscope fitted with Plan-neofluar oil-immersion objectives, a 100 W Hg source and Chroma #8300 triple band pass filter set. Image capture and analysis of nuclear size and distance between the centroids of the hybridization signals was performed with scripts written for IPLab Spectrum (Scanalytics Copr, Fairfax, VA) as previously described [67]. Three-dimensional images were captured at 200 nm intervals in the z axis, using an objective fitted with a Pifoc motor.

Images from 30–50 nuclei were analysed in each experiment and the significance of any difference in the data distributions was assessed using the non-parametric Mann-Whitney U test. A p-value ≤0.05 was considered statistically significant.

### Western blotting

The cells were washed with ice-cold PBS and total cell lysates were prepared by resuspending the cells in lysis buffer. The samples were boiled for 20 min at 95°C and cleared by centrifugation at 12 000×*g* for 10 mins. Next, the samples were subjected to SDS-PAGE and the proteins transferred onto nitrocellulose membrane. Western blot analysis was performed using ERα (HC-20, Santa Cruz Biotechnology, Inc.) and GAPDH (MAB374, Chemicon International) antibodies and processed using the MultiGauge Software from FUJI.

### qRT–PCR

Total RNAs were extracted using TRIzol Reagent (Invitrogen). 1–5 µg of total RNA was reverse transcribed in a final volume of 20 µl using SuperScriptr III Reverse Transcriptase. cDNA was stored at −80°C. All target transcripts were detected using quantitative RT-PCR (SYBRGreen) assays on a Mastercycler Realplex device using *RPLP0* as endogenous control for normalization of the data. The following primer pairs were used for amplification:

*RPLP0*: (Fwd) 5′-TGGCAGCATCTACAACCCTGAA -3′

(Rev) 5′-ACACTGGCAACATTGCGGACA -3′

*GREB1*: (Fwd) 5′-GTGGTAGCCGAGTGGACAAT-3′

(Rev) 5′-AAACCCGTCTGTGGTACAGC-3′

*TFF1:* (Fwd) 5′-CCCCTGGTGCTTCTATCCTAAT-3′

(Rev) 5′-CAGATCCCTGCAGAAGTGTCTA-3′

*PGR:* (Fwd) 5′-CTTAATCAACTAGGCGAGAG-3′

(Rev) 5′-AAGCTCATCCAAGAATACTG-3′

*CTSD:* (Fwd) 5′-GCGAGTACATGATCCCCTGT-3′

(Rev) 5′-CTCTGGGGACAGCTTGTAGC-3′

The thermal cycling condition comprised 2 mins at 50°C and 2 mins at 95°C followed by 40 PCR cycles (95°C for 15 sec, 58°C for 30 sec, 72°C for 20 sec). Melting curves were recorded from 60°C to 95°C and all PCR products revealed single bands. The results were analyzed using Mastercycler Realplex and qBASE software.

## Supporting Information

Figure S1 Localisation of signals for *TFF1* and *GREB1* on metaphase chromosomes and in nuclei from HT1080 and MCF10A cells. (A) FISH with probes for *TFF1* (red) and *GREB1* (red) and paints for chromosomes 2 and 21 respectively (green) on metaphase chromosome spreads of HT1080 cells. (B) Interphase FISH with probes for *TFF1* (green) and *GREB1* (red) on nuclei from HT1080 cells. (C) FISH with probes for *TFF1* (red) and *GREB1* (green) and paints for chromosomes 2 (red) and 21 (green) respectively on metaphase chromosome spreads of MCF10A cells. (D) Interphase FISH with probes for *TFF1* (red) and *GREB1* (green) on nuclei from MCF10A cells.
(5.60 MB TIF)

Figure S2 Nuclear organisation of *TFF1* and *GREB1* in MCF-7 cells and their derivatives. (A) Box plots show inter-probe distances (d) normalized to nuclear radius (r) between homologous or heterologous alleles, as measured either by 3D FISH in nuclei of MCF-7 cells grown in the absence of E2 (-E2) or 1 and 16 hr after the addition of 100 nM E2. Asterisks indicate data points beyond the 95th percentile. N = 50 cells. (B) Box plots showing inter-probe distances (d) normalized to nuclear radius (r) between heterologous *TFF1* and *GREB1* alleles, as measured by 2D FISH in nuclei of LCC1 or LCC9 cells grown in the absence of E2 (-E2) or after 1 hr in the presence of 100 nM E2. Shaded boxes show the mean and 25–75 percentile of the data. Asterisks indicate data points beyond the 95th percentile. N = 50 cells.
(1.16 MB TIF)
