# Supplementary figures and images for: Activation of Estrogen-Responsive Genes Does Not Require Their Nuclear Co-Localization
Source: PLoS Genet. 2010 Apr 22;6(4):e1000922. doi: 10.1371/journal.pgen.1000922 (PMC2858706; doi:10.1371/journal.pgen.1000922)

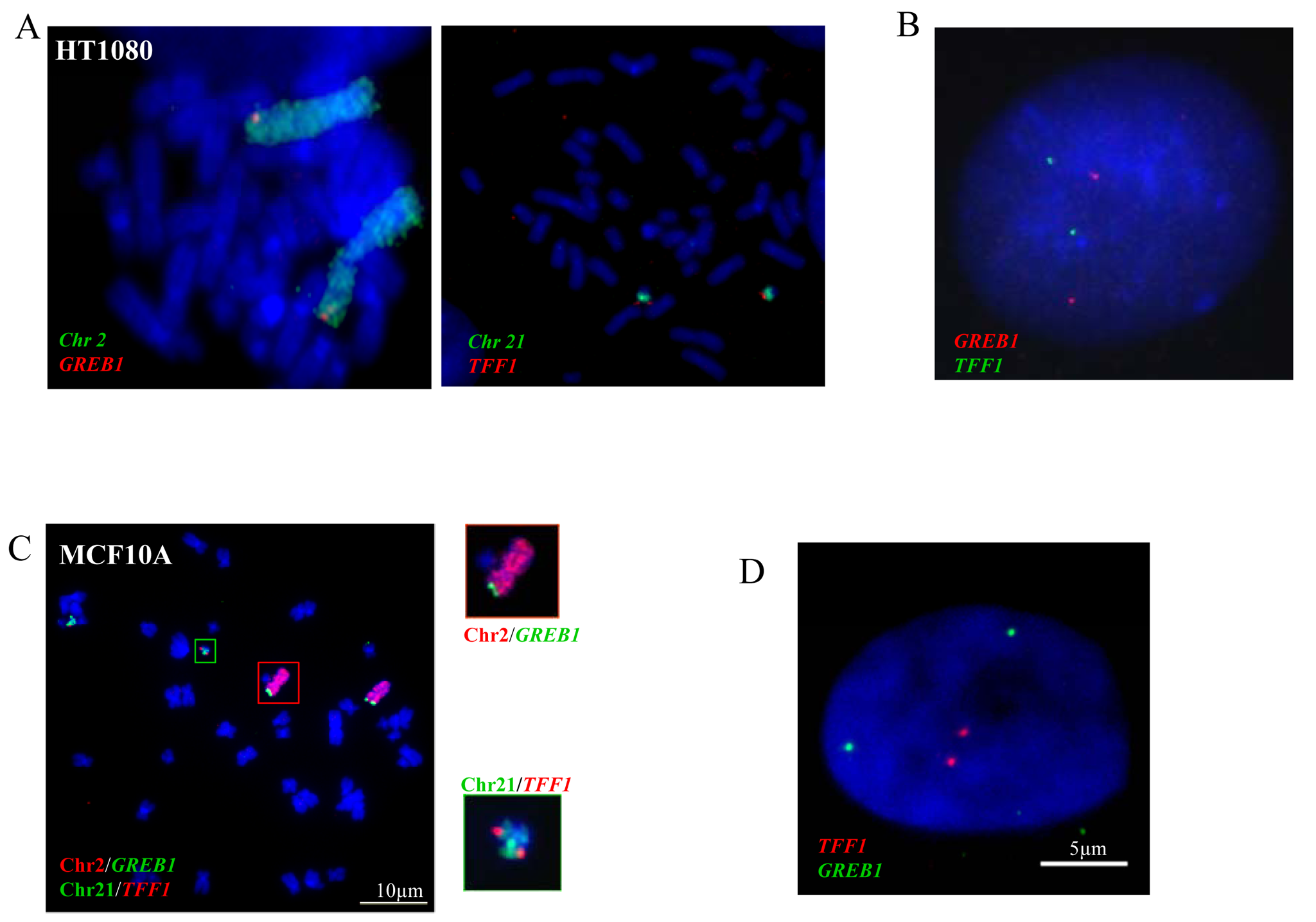

Supplement: Figure S1 — Localisation of signals for TFF1 and GREB1 on metaphase chromosomes and in nuclei from HT1080 and MCF10A cells. (A) FISH with probes for TFF1 (red) and GREB1 (red) and paints for chromosomes 2 and 21 respectively (green) on metaphase chromosome spreads of HT1080 cells. (B) Interphase FISH with probes for TFF1 (green) and GREB1 (red) on nuclei from HT1080 cells. (C) FISH with probes for TFF1 (red) and GREB1 (green) and paints for chromosomes 2 (red) and 21 (green) respectively on metaphase chromosome spreads of MCF10A cells. (D) Interphase FISH with probes for TFF1 (red) and GREB1 (green) on nuclei from MCF10A cells. (5.60 MB TIF) [file pgen.1000922.s001.tif]

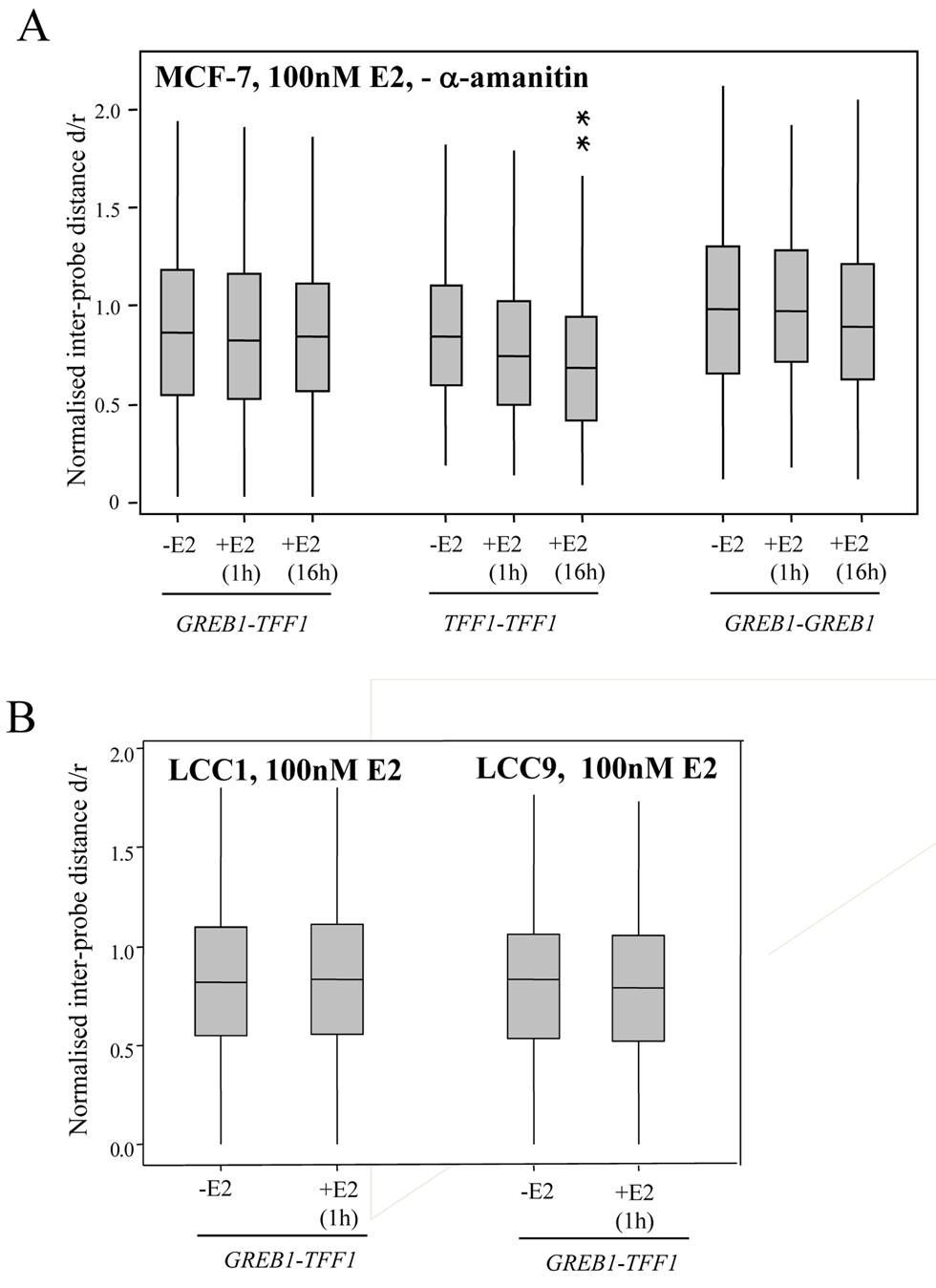

Supplement: Figure S2 — Nuclear organisation of TFF1 and GREB1 in MCF-7 cells and their derivatives. (A) Box plots show inter-probe distances (d) normalized to nuclear radius (r) between homologous or heterologous alleles, as measured either by 3D FISH in nuclei of MCF-7 cells grown in the absence of E2 (-E2) or 1 and 16 hr after the addition of 100 nM E2. Asterisks indicate data points beyond the 95th percentile. N = 50 cells. (B) Box plots showing inter-probe distances (d) normalized to nuclear radius (r) between heterologous TFF1 and GREB1 alleles, as measured by 2D FISH in nuclei of LCC1 or LCC9 cells grown in the absence of E2 (-E2) or after 1 hr in the presence of 100 nM E2. Shaded boxes show the mean and 25–75 percentile of the data. Asterisks indicate data points beyond the 95th percentile. N = 50 cells. (1.16 MB TIF) [file pgen.1000922.s002.tif]
